# Immortalization of human AE pre-leukemia cells by hTERT allows leukemic transformation

**DOI:** 10.18632/oncotarget.11093

**Published:** 2016-08-05

**Authors:** Shan Lin, Junping Wei, Mark Wunderlich, Fu-Sheng Chou, James C. Mulloy

**Affiliations:** ^1^ Cancer and Blood Disease Institute, Cincinnati Children's Hospital Research Center, Cincinnati, OH, USA

**Keywords:** immortalization, hTERT, t(8;21) fusion genes, human HSPC

## Abstract

Human CD34+ hematopoietic stem and progenitor cells (HSPC) expressing fusion protein AML1-ETO (AE), generated by the t(8;21)(q22;q22) rearrangement, manifest enhanced self-renewal and dysregulated differentiation without leukemic transformation, representing a pre-leukemia stage. Enabling replicative immortalization via telomerase reactivation is a crucial step in cancer development. However, AE expression alone is not sufficient to maintain high telomerase activity to immortalize human HSPC cells, which may hamper transformation. Here, we investigated the cooperativity of telomerase reverse transcriptase (hTERT), the catalytic subunit of telomerase, and AE in disease progression. Enforced expression of hTERT immortalized human AE pre-leukemia cells in a telomere-lengthening independent manner, and improved the pre-leukemia stem cell function by enhancing cell proliferation and survival. AE-hTERT cells retained cytokine dependency and multi-lineage differentiation potential similar to parental AE clones. Over the short-term, AE-hTERT cells did not show features of stepwise transformation, with no leukemogenecity evident upon initial injection into immunodeficient mice. Strikingly, after extended culture, we observed full transformation of one AE-hTERT clone, which recapitulated the disease evolution process in patients and emphasizes the importance of acquiring cooperating mutations in t(8;21) AML leukemogenesis. In summary, achieving unlimited proliferative potential via hTERT activation, and thereby allowing for acquisition of additional mutations, is a critical link for transition from pre-leukemia to overt disease in human cells. AE-hTERT cells represent a tractable model to study cooperating genetic lesions important for t(8;21) AML disease progression.

## INTRODUCTION

Chromosome translocation t(8;21)(q22;q22) generating the AML1-ETO (AE) fusion protein, is one of the most frequent genetic aberrations in acute myeloid leukemia (AML), accounting for 10% of all disease [1]. AE is the driver oncogene, and expression of AE in hematopoietic stem and progenitor cells (HSPC) results in dysregulation of differentiation and enhancement of self-renewal. Yet both murine and human cell based models have demonstrated that HSPC expressing AE alone are only able to establish a pre-leukemia state without overt disease, and full transformation requires additional cooperating mutations [2-4]. AML induction by co-expression of AE with a cooperating mutant is successfully demonstrated in murine cells [5-7]. However, an AE leukemia model derived from human HSPC is not yet available. Attempts by introduction of NRAS(G12D), KIT or CBL mutant into AE-expressing human CD34+ HSPC (AE cells) failed to initiate AML in immunodeficient mice [8-10], suggesting that human HSPC are more resistant to oncogene-induced transformation and other critical cooperating factors are required.

Enabling replicative immortality is regarded as one hallmark of cancer [11]. AE pre-leukemia cells can be detected in patients decades before disease onset or after complete remission, suggesting these cells have acquired unlimited proliferative capacity [12, 13]. Though expression of AE can dramatically prolong the lifespan of human HSPC compared to normal cells *in vitro*, the AE cells are not immortalized, ceasing proliferation after about 25-30 weeks *in vitro*, even when other introduced mutations are present [4, 8, 10]. Thus, replicative immortality could be one missing link for AE cells to evolve from a pre-leukemia state to full transformation.

One critical mechanism by which cancer cells become immortalized is reactivation of telomerase, a specialized reverse transcriptase that is involved in the synthesis of telomere at the chromosomes ends and is active in the majority of human tumors but is not expressed in most non-immortalized somatic cells [14]. Telomerase stops telomere erosion and prevents cell cycle arrest, senescence and apoptosis initiated by telomere attrition to a critically short length [15]. In several types of somatic human cells, such as T cells, fibroblasts and endothelial cells, constitutive hTERT expression is sufficient to promote immortalization [16-19]. In addition, hTERT can enhance stem cell activity and facilitate oncogene-induced transformation *via* functions beyond telomere maintenance, including promoting cell proliferation, reducing DNA damage and increasing cell survival [20, 21]. On the other hand, ablating telomerase activity is reported to impair cell growth and disease progression of several hematopoietic malignancies, including AML [22-24].

Therefore, we hypothesized that enhanced telomerase activity would endow AE pre-leukemia cells with limitless replicative potential and promote disease progression. In the present study, we investigated the biological consequence of forced expression of hTERT in AE pre-leukemia cells by retroviral transduction.

## RESULTS

### Expression of hTERT in AE pre-leukemia cells results in immortalization

**Figure 1:**
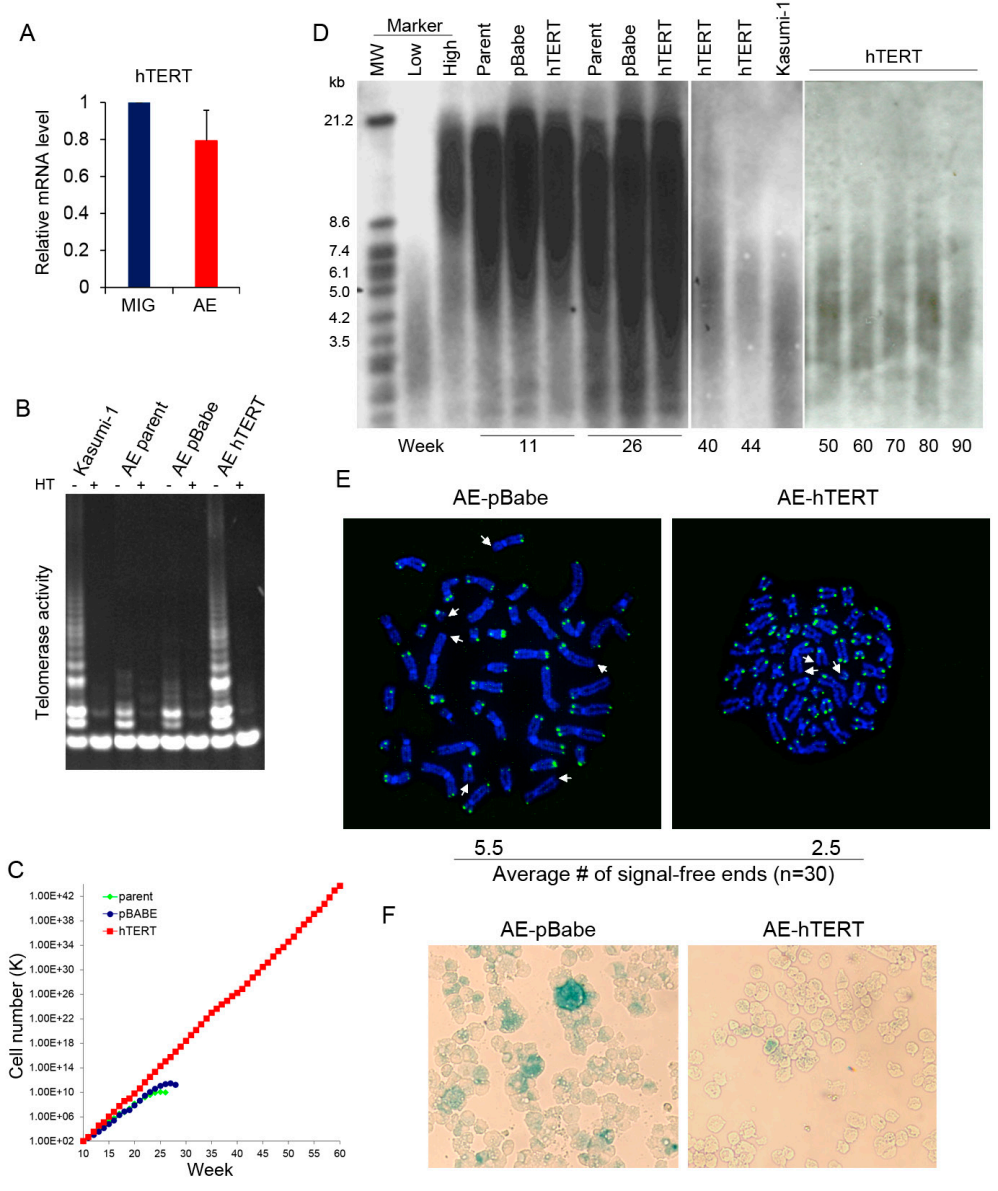
AE pre-leukemia cells are immortalized by hTERT **A.** hTERT mRNA analyzed by qPCR in CD34+HSPC transduced with AE or control empty vector (MIG). Error bar represents SD, *n* = 4. **B.** Telomerase activity of control AE, AE-hTERT and Kasumi-1 cells. Cell extracts heated (HT) to inactivate telomerase were used as negative control. **C.** Weekly cell count of AE-hTERT and control AE cells. **D.** Telomere length of AE-hTERT and control cells from culture of different time points measured by southern blot with a telomeric probe. **E.** Telomere FISH analysis by telomere specific DNA probe on week 26 AE-hTERT and AE-pBabe cells. Representative cells at metaphase are shown, telomere-free chromosome ends are indicated by arrow. 30 metaphases for each sample were scored, and average number of telomere-free chromosome ends were indicated (*p* < 0.01, two-tailed *t*-test). **F.** β-Galactosidase staining shows AE-pBabe cells have increased senescence compared to AE-hTERT cells at week 26. One representative of two independent experiments is shown.

Previously we have reported that AE cells showed only a low level of telomerase activity that was not sufficient to confer immortality [4]. Indeed, transduction of AE in human CD34+ HSPC did not result in upregulation of hTERT compared to HSPC transduced with control empty vector (Figure 1A). The telomerase activity in AE cells was much lower than levels seen in the immortal AML cell line Kasumi-1 derived from a t(8;21) patient (Figure 1B). To achieve a higher telomerase activity, AE cells were transduced with the retrovirus expressing hTERT (AE-hTERT), or with a control empty vector (AE-pBabe). Independent AE clones stably expressing hTERT or pBabe were selected through puromycin resistance. Telomerase activity was upregulated in AE-hTERT cells, becoming comparable to the levels in Kasumi-1 cells. In contrast, control vector transduced AE cells did not show a significant change in telomerase activity (Figure 1B). While control cells grew at a rate of about 2 population doublings per week and stopped proliferating at around week 26, AE-hTERT cells showed continuous proliferative capacity at an enhanced rate of about 2.5 population doublings a week (Figure 1C). Therefore, enforced expression of hTERT led to immortalization of AE pre-leukemia cells.

Interestingly, the immortalization of AE cells was not associated with telomere lengthening. Despite upregulated telomerase activity, AE-hTERT cells showed a progressive decline in telomere length similar to control cells. The telomere length of AE-hTERT cells was eventually stabilized at about 3-4kb, comparable to the telomere length of Kasumi-1 cells (Figure 1D). This telomere elongation-independent lifespan extension has been reported in other cell types [25, 26]. It has been suggested that the “cap” function of hTERT contributes to this phenotype, by which hTERT with other telomere-binding proteins form a “telomere cap” to prevent the exposure of chromosome ends, and thus the initiation of senescence [27]. Accordingly, we performed telomere FISH on cell cultures at week 26, when control cells were losing proliferative capacity, and found a significantly lower number of telomere-free ends in AE-hTERT cells compared to AE-pBabe cells (Figure 1E). Additionally, AE-pBabe but not AE-hTERT cells manifested increased senescence indicated by SA-β-Galactosidase staining (Figure 1F). These results demonstrate that hTERT expression in AE cells maintained telomere integrity and prevented senescence induced by telomere crisis. This hTERT-mediated immortalization of AE cells was independent of excessive telomere lengthening.

### hTERT improves stem cell function of AE pre-leukemia cells

**Figure 2:**
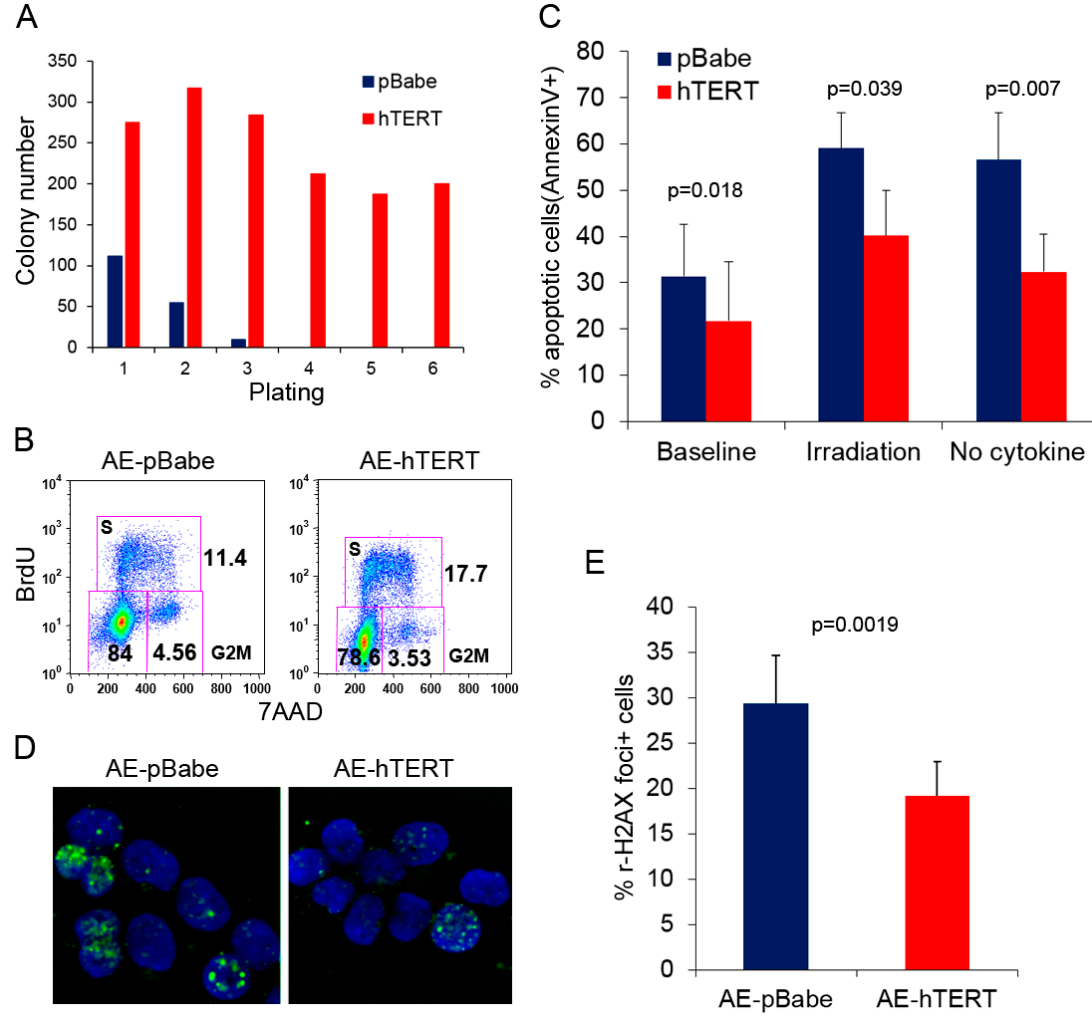
hTERT improves stem cell function of AE pre-leukemia cells **A.** Colony replating assay for AE-hTERT and AE-pBabe cells. 10,000 cells were seeded for each plating in triplicate. Results represent mean. One representative of three independent experiments is shown. **B.** Representative cell cycle analysis shows AE-hTERT cultures contain more cells in S-phase than AE-pBabe cell cultures. **C.** Apoptosis analysis measured by Annexin V staining on AE-hTERT and AE-pBabe cells under indicated conditions. Results represent mean +/− SD. p value was calculated by two-tailed paired student *t*-test, *n* = 5. **D.** Immunostaining for H2AX phosphorylation (Ser 139, green) in AE-hTERT and AE-pBabe cells. DNA was counterstained with DAPI (blue). **E.** Quantification results of **D.**, representing mean +/− SD. p value was calculated by two-tailed paired *t*-test, *n* = 5.

AE pre-leukemia stem cells are enriched in the CD34+ fraction [4]. The percentage of CD34+ cells was not significantly different between hTERT-expressing and control AE cells, ranging from 10-30% (data not shown), suggesting that hTERT did not affect the size of the phenotypic stem cell pool. hTERT overexpression has been shown to improve stem cell function of embryonic and somatic stem cells [20, 28]. To test whether hTERT can also improve the function of AE pre-leukemia stem cells, we sorted CD34+ cells from AE-hTERT and pre-senescent control AE cells (weeks 15-18) and performed methylcellulose colony replating assay, using clonogenic capacity as a surrogate readout of stem cell function *in vitro*. In the first plating, a 3 fold increase in colony number was observed in hTERT-expressing AE cells when compared to control AE cells (Figure 2A). Cells were collected for replating every 2 weeks. Strikingly, the hTERT-expressing cells were highly successful in their continuous replating, with sustained clonogenic ability for up to 6 platings. By comparison, control cells lost replating activity after the third plating (Figure 2A). These results show that hTERT enhances the stem cell activity of AE cells.

hTERT can improve stem cell function *via* influencing multiple aspects of cell physiology [29]. Thus we investigated the cellular mechanisms accounting for the hTERT-mediated enhancement of AE stem cell function. Since AE-hTERT cells underwent 0.5 extra population doubling every week compared to control cells (Figure 1C), this suggests that hTERT promoted cell proliferation and/or survival. Indeed, an increase in S phase cells was detected in AE-hTERT-expressing cells compared to control cells by bromodeoxyuridine (BrdU) incorporation staining (Figure 2B). Next, we examined cell apoptosis under physiological conditions or after stimulation by irradiation or cytokine withdrawal. The levels of both basal and induced apoptosis were significantly lower in AE-hTERT cultures, suggesting that expression of hTERT protected cells from cell death (Figure 2C). Immunostaining analysis of phosphorylated histone H2AX, a marker for DNA damage foci, showed that AE-hTERT cells had lower levels of DNA damage compared to AE-pBabe cells (Figure 2D and 2E), implying that either an attenuated DNA damage response or increased kinetics of DNA damage repair accounts for the reduced apoptosis of AE-hTERT cells. Taken together, these data suggest that the hTERT promotes AE stem cell function by increasing proliferation and enhancing survival.

### hTERT does not cause stepwise transformation directly

**Figure 3:**
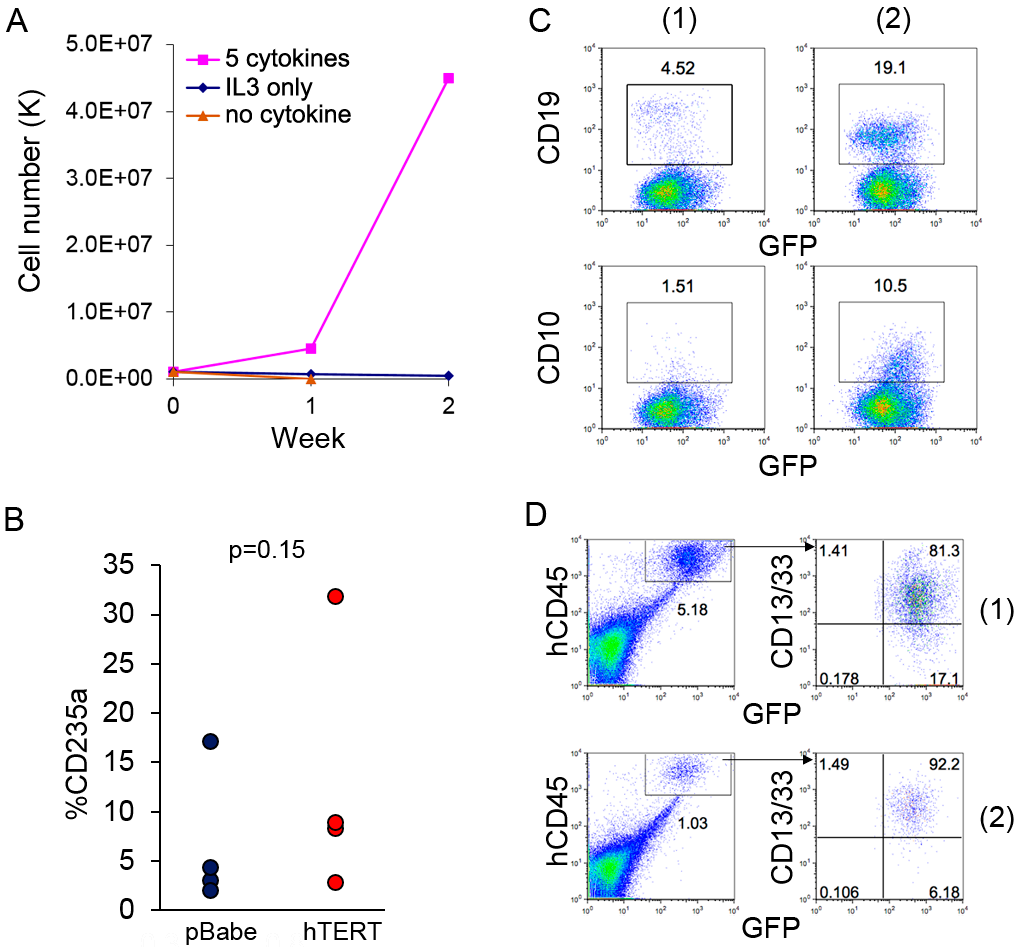
Expression of hTERT does not lead to direct leukemia transformation of AE cells **A.** Growth curve of AE-hTERT cells in culture with indicated cytokines demonstrates AE-hTERT cells did not achieve cytokine independence. **B.** Percentage of CD235a+ cells of AE-hTERT and AE-pBabe cells from first plating of colony forming assay shows that AE-hTERT cells kept similar erythroid lineage potential as AE-pBabe cells. p value was calculated by two-tailed *t*-test. **C.** Flow cytometry analysis demonstrates that AE-hTERT cultures are able to produce CD19+ and CD10+ lymphoid cells under B-cell differentiation condition. 2 independent experiments are shown. **D.** Flow cytometry analysis of murine BM at 16 weeks post-transplantation indicates AE-hTERT cells maintain myeloid (CD13/33+) engraftment in immunodeficient mice. Human cell engraftment was indicated by GFP+hCD45+; two representative mice are shown.

Parental AE cells are strictly cytokine-dependent, and though the cells are largely myeloid-lineage biased, they still retain limited erythroid- and lymphoid-lineage potential [4]. Though not leading to AML, introduction of cooperating mutation, such as NRas(G12D), can promote progression of AE cells toward transformation, with features including cytokine independence and block of erythroid-lineage potential in methylcellulose colony assay [8]. We investigated whether hTERT expression induced features of stepwise transformation. AE-hTERT cells cannot be maintained in conditions with no cytokine or IL-3 only, indicating expression of hTERT did not result in cytokine independence (Figure 3A). In the methylcellulose colony assay, we did not observe a significant difference in the percentage of erythroid cells between AE-hTERT and AE-pBabe as measured by CD235a staining (Figure 3B), demonstrating the erythroid potential of AE-hTERT cells was not changed. Additionally, similar to what we have reported for parent AE cells [4], when cultured on MS-5 stroma with lymphoid cytokine supplementation, AE-hTERT cultures were able to generate lymphoid cells expressing CD19 and CD10 (Figure 3C). These data suggest that expression of hTERT did not alter the multi-lineage competence of AE cells.

We next determined whether immortalized AE-hTERT cells were able to initiate AML *in vivo*. 10 million AE-hTERT cells (15-20 weeks in culture) were injected into immunodeficient mice. No AML symptoms were observed in recipient mice. To confirm the presence of engrafted cells, mouse bone marrow was analyzed 4 months after transplantation. We found low level engraftment of human cells in recipient mouse bone marrow, which were exclusively myeloid as shown by CD13/CD33 positivity (Figure 3D). Therefore, hTERT cannot readily induce stepwise transformation of AE cells. Thus, although AE-hTERT cells can maintain a long-term graft *in vivo*, they are not sufficient to cause AML.

### Extended culture allows mutation accumulation and transformation of AE-hTERT cells

**Figure 4:**
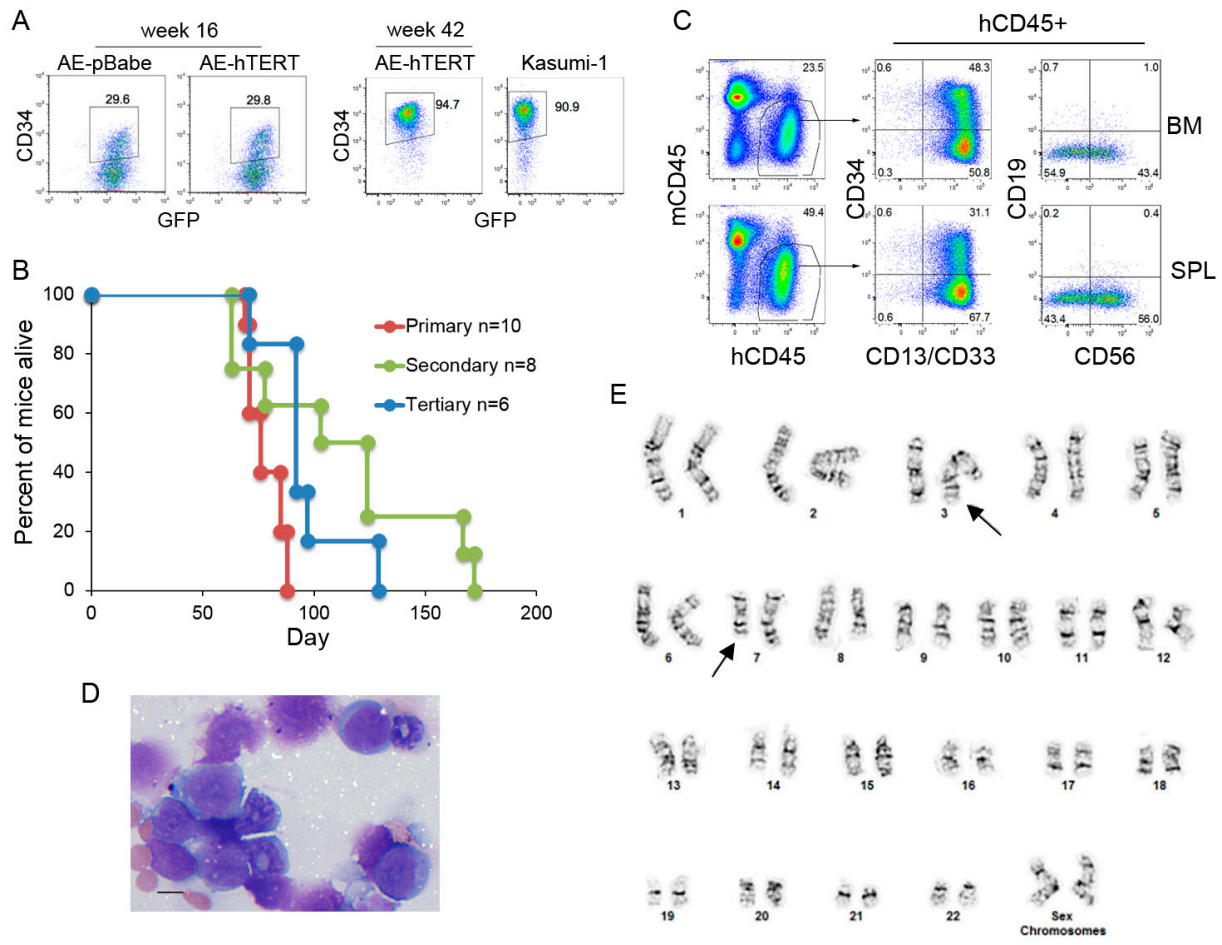
Leukemic transformation is observed on extended culture of AE-hTERT cells **A.** Flow cytometry analysis for CD34 expression on AE-hTERT clones at early and late passages. AE-pBabe and Kasumi-1 cells were used for comparison. **B.** Kaplan-Meier survival curve of recipient mice. **C.** Flow cytometry analysis showed the malignant cells infiltrating BM and spleen are of myeloid lineage with CD34 and CD56 expression. **D.** Wright-Giemsa-stained BM cytospins showed the presence of malignant myeloid blast cells. Scale bar = 10 um. **E.** Spectral karyotype of leukemic AE-hTERT cells showing chromosome translocation t(3;7)(q27;q22) (arrows).

Previous studies have shown that cells expressing AE have an increased DNA mutation frequency [30, 31]. Therefore, although hTERT expression did not lead to direct transformation of AE cells, the prolonged lifespan of AE-hTERT cells could increase the chance to accumulate functional mutations for disease progression. Interestingly, after extended culture for more than 40 weeks, one AE-hTERT clone showed an abnormally high percentage of CD34+ cells compared to the cells at an early stage, similar to CD34 levels seen in Kasumi-1 cells (Figure 4A). As this change may represent a progression to transformation, the leukemogenicity of these cells was tested in mice. Although the parental clone and the early passage of this AE-hTERT clone were unable to initiate AML as shown for other tested AE-hTERT clones (Figure 3D and data not shown), excitingly, all immunodeficient mice receiving the CD34hi AE-hTERT cells got sick between 60-100 days after transplantation (Figure 4B). High infiltration of human cells was confirmed in spleen and bone marrow. The engrafted cells showed a myeloid phenotype and retained increased expression of CD34 (Figure 4C). Since t(8;21) leukaemia is frequently associated with aberrant expression of the lymphoid antigens, CD19 and CD56, we therefore examined whether these antigens were expressed on the engrafted cells [32]. Positive CD56 expression was detected but there was no definitive CD19 expression (Figure 4C). Morphological analysis validated the presence of myeloid blasts (Figure 4D). Importantly, the disease could be transferred into secondary and tertiary recipients (Figure 4B), confirming the malignant nature of the disease and thus the full transformation of this AE-hTERT clone. Although fully transformed, this AE-hTERT clone did not achieve cytokine independency (Figure S1A). Previous studies have shown that AE upregulates MPL, the receptor for thrombopoietin(TPO), and the long-term growth of AE cells depends on TPO/MPL signaling [33, 34]. Consistent with this finding, TPO withdrawal from the culture medium reduced the proliferation of the transformed AE-hTERT clone (Figure S1B), suggesting this clone still relies on TPO/MPL signaling and likely other AE-mediated molecular programs for growth. To test for potential cooperating genetic aberrations, cytogenetics of the leukemic cells was analyzed, showing a karyotype of t(3;7)(q27;q22) (Figure 4E). Intriguingly, this chromosomal translocation has also been identified in the complex cytogenetic AML patient-derived cell line Kasumi-3 [35]. Although the functional significance of this mutation in cooperating transformation remains to be tested, this result demonstrates that the proliferative immortalization endowed by hTERT activation serves as a critical link in AE AML evolution.

## DISCUSSION

AE cannot fully transform HSPC by itself, although it is the critical initiating oncogene and is required for leukemia maintenance. In agreement with this finding, t(8;21) AML was reported to associate with a high number of additional genetic lesions [36]. Interestingly, while several murine AE AML models have been established by co-expression of AE with another mutant gene, this approach has not been successful when using human cells. This suggests that more than one cooperating event may be required to generate human AE AML. Targeted sequencing shows that about half of t(8;21) AML patient samples harbored more than one mutation, and it is likely a comprehensive whole-genome sequencing effort will reveal most patients do so [37]. Acquiring multiple additional mutations could be a slow process. Since AE pre-leukemia cells can be detected years before disease onset [12], it is likely AE pre-leukemia cells have long-term self-renewal or unlimited replicative capacity to allow mutation accumulation and transformation within an extended time span. We and others have reported that AE expression can promote mutagenesis [30, 31], however, disease transformation of AE pre-leukemia cells within the usual lifespan of the cell cultures has never been reported, indicating that the relatively short lifespan is not sufficient for accumulation of functional mutations and clonal evolution. In contrast, in this study, we found one AE-hTERT clone achieved full transformation after extended culture, possibly reflecting the disease progression in patients, and highlighting the critical role of replicative immortality in AML development. For unknown reasons, both Kasumi-1 cell line and t(8;21) patient samples engraft poorly in immunodeficient mice [38], and there have been no reports on an efficient human xenograft model to study t(8;21) AML. This transformed AE-hTERT cell line successfully induced AML in immunodeficient mice with high infiltration in mouse bone marrow and spleen, and can serve as a useful preclinical model for AE AML.

A previous study has shown that hTERT by itself cannot immortalize human CD34+ HSPC [39], suggesting that the long-term proliferation of AE-hTERT cells still depends on self-renewal signals promoted by AE. Of note, increased hTERT expression did not lead to direct transformation of AE cells, as opposed to some other tumor models reported [21]. AE-hTERT cells retained many physiological features found in parental AE cells, such as multi-lineage differentiation potential and cytokine dependency. Therefore, it is unlikely that hTERT dramatically changes the AE molecular signaling network. Given the advantage of robust long-term growth, AE-hTERT cells are a more tractable model compared to parental AE cells for studying the pre-leukemia stage of t(8;21) AML. AE-hTERT cells could be a valuable tool for investigating the functions of recurrent mutations identified in t(8;21) AML patients, and screening for critical functional components and drug targets for AE molecular programs. Interestingly, a rare recurrent chromosome translocation identified in hematological malignancy, t(3;7)(q27;q22), was found in the transformed AE-hTERT cells. According to the Cancer Genome Anatomy Project (CGAP) database (cgap.nci.nih.gov), several abnormalities related to chromosome 3q27, frequently found in lymphoma, involve the *BCL6* gene, which can prevent TP53 signaling in response to DNA rearrangements [40]. Additionally, chromosome alterations in the nearby region 3q26, including t(3;7)(q26;q21), often occur in AML. 3q26 rearrangements result in aberrant upregulation of *EVI1*, which is a known oncogene in AML [41, 42]. Nevertheless, whether *BCL6* or *EVI1* may collaborate with AE in leukemic transformation is unclear, and their potential role in this particular AE-hTERT clone requires future study. In addition, we cannot rule out other possible gene mutations that are not reflected by karyotype analysis. Full characterization of the genetic lesions found in this AE-hTERT clone will provide insights on cooperating events required for t(8;21) AML.

Our study suggests that upregulation of hTERT and telomerase activity could be an important cooperating event for t(8;21) AML development. However, how hTERT is activated in t(8;21) patients is unclear. It was reported that the AE protein itself is required for maintaining hTERT expression in Kausmi-1 cells [43], whereas we showed that hTERT was not upregulated upon AE expression in CD34+ HSPC and AE cells did not maintain high telomerase activity, suggesting that the regulation of hTERT by AE may be cell-context dependent. It is possible that AE alone is not sufficient to activate hTERT at the initial stage of leukemia, but collaborates with other genetic events in leukemic cells to upregulate hTERT during progression. Wnt/beta-catenin signaling was also reported to regulate hTERT [44], however beta-catenin is activated in HSPC expressing AE [45], implying that Wnt/beta-catenin signaling by itself is not able to upregulate hTERT in HSPC. Alternatively, recent studies in solid tumors have revealed that point mutations in the hTERT promoter can increase promoter activity and thus hTERT expression [46]. Whether this mechanism is utilized by t(8;21) AML needs further investigation.

Forced expression of hTERT in AE cells did not result in telomere lengthening. It is likely that hTERT-mediated immortalization of AE cells is mainly through maintaining telomere integrity and repressing replicative senescence. In addition to conferring immortalization, hTERT also improved stem cell function of AE cells by increasing cell proliferation and survival, guaranteeing the robust long-term growth. hTERT also attenuated DNA damage induced apoptosis, which could increase the tolerance of AE cells to genome instability and contribute to the final transformation. Interestingly, this high hTERT activity together with shortened telomere have been identified in several types of hematopoietic malignancies including AML [47, 48]. Therefore, AML cells could be particularly sensitive to telomerase inhibition compared with normal HSPC, since their telomeres are already at critically short lengths and their proliferation and survival may also depend on hTERT activity. The efficacy of telomerase inhibitors for targeting AML has been validated in preclinical experiments [24, 49]. And intriguingly, other targeted AML therapies have been shown to elicit effects partially through telomere dysregulation in AML cells [50]. Our study showing that telomerase activity induction appears to be crucial for AE pre-leukemia stem cell maintenance and disease progression provides additional evidence to support that telomerase inhibition can be a potential therapy for AML treatment.

## MATERIALS AND METHODS

### Cell culture

AE pre-leukemia cells were established as previous describe [4], briefly, CD34+ HSPC cells were purified from human umbilical cord blood cells (CB) or adult peripheral blood progenitor cells (PBPC), and transduced with retrovirus enveloping MSCV-AE-IRES-GFP. AE cells were cultured in IMDM supplemented with 20% BIT 9500 serum substitute (Stemcell Technology) and cytokine cocktail of SCF (Stem cell factor), TPO (Thrombopoietin), Flt3 Ligand, IL-6 and IL-3 (all at 10ng/mL) (Peprotech).

### Retrovirus production and transduction

Retoviral vector expressing hTERT (pBabe-puro-hTERT) and control empty vector (pBabe-puro) were obtained from Dr. Robert Weinberg (Addgene). To produce retrovirus, 1.5ug of each construct was transfected respectively into phoenix-gp cells on 6 well plates along with pSV-ampho-env (1.0ug) and pEQ-PAM3(-E) (0.5ug) using TransIT reagent (Mirus). Supernatant was collected at 36 and 48 hours. CD34+ cells were selected from week 6-8 old AE pre-leukemia cultures using EasySep human CD34 selection kit (Stemcell Technology). Retronectin (Takara Shuzo) coated plates were preloaded with the virus supernatant by spinoculation at 2000 g for 2hr at 22°C, and then loaded with CD34+ AE cells suspended in fresh viral supernatant. 8 hours later, transduced cells were harvested, expanded for 2 days, and then selected with 0.4ug/ml puromycin for 1 week.

### Telomerase activity assay

Cellular extracts were prepared in 1x CHAPS lysis buffer and cleared by centrifuge at 12,000 g and 4°C for 20 min. Telomerase activity was assayed with a PCR-based telomeric repeat amplification protocol (TRAP) using telomerase detection kit (Intergen). 25ul of the reaction products were resolved on an 8-16% polyacrylamide gel.

### Methylcellulose colony-forming assay

Assays were performed using MethoCult H4100 medium (StemCell Technologies)** supplemented with 20% BIT, 50uM β-Mercaptoethanol, 2mM L-glutamine, 100U/mL penicillin/streptomycin, and the human cytokines Erythropoietin (EPO, 6U/mL), Granulocyte Colony Stimulating Factor (G-CSF, 10ng/mL), IL-6(20ng/mL), IL-3 (20ng/mL), and SCF (20ng/mL) (Peprotech). Methocult containing 1×10^4^ cells was dispensed into 35-mm dishes. Colonies of greater than 50 cells were scored at day 14. Colonies were collected and 1×10^4^ cells were used for replating. Collected cells were stained with PE-CD235a (BD Biosciences) for flow cytometry analysis to evaluate erythroid-lineage potential.

### Cell cycle and apoptosis analysis

Cell cycle was analyzed by BrdU incorporation assay using APC-BrdU flow kit (BD Bioscience), cells were co-stained with 7-AAD. For apoptosis analysis, non-treated cells and cells exposed to 10 Gy irradiation or 24-hour cytokine depletion were measured using the PE-annexinV apoptosis detection kit (BD Bioscience) by flow cytometry.

### B cell differentiation

AE-hTERT cells were cultured on a monolayer of MS-5 stroma cells in aMEM containing 10%FBS and 10ng/mL each of SCF, Flt3L, and IL-7 (Peprotech). Cultures were demi-depopulated weekly and tested for B cell differentiation 4 weeks later by staining with antibodies for APC-CD19 and PE-CD10 (BD Bioscience).

### Xenograft transplantation

6- to 8-week-old NSGS mice (Jackson Labs) were conditioned with sublethal irradiation 8 h before transplantation, or 30mg/kg busulphan (Sigma) through intraperitoneal injection 24 h before transplantation. 500,000 AE-hTERT cells were transplanted *via* intrabone injection into NSGS mice. Mouse bone marrow was examined by aspiration at indicated time points, and mice were sacrificed when signs of illness were observed. Organs were homogenized and processed for flow cytometry analysis, human cells were identified by GFP expression and APC- or PECy7-human CD45 staining (BD Bioscience). Other surface markers analyzed include APCCy7-mouse CD45, APC-CD34, PE-CD33, PE-CD13, BV510-CD56(BD Bioscience) and VioBlue-CD19 (Miltenyi Biotec). In serial transplantation, 500,000 bone marrow or spleen cells were injected through tail vein into unconditioned NSGS mice.

### Southern blot analysis for telomere length

2ug genomic DNA was digested with restrict enzymes Hinf1 and RsaI (Roche) at 37°C overnight. The DNA fragments were resolved by 0.8% agarose gel electrophoresis at 60v for 8h, transferred to a nylon membrane (Osmonics) in 20XSSC and hybridized with the telomeric probe (TTAGGG)_4_ as previously described [51].

### Telomere FISH and karyotype

Cells were treated with colcemid arrest (0.16ug/ml, Invitrogen) overnight at 37°C followed by hypotonic solution containing 0.075M KCl for 15 min at 37°C and then fixed with cold 3:1 Methanol:Acetic Acid fixative. Cells were washed with fixative six times and then dropped onto clean slides. Slides were chilled at 4°C overnight and then used for karyotype analysis and hybridization for Q-FISH with a Cy3-(CCCTAA)3 PNA telomere probe (Applied Biosystems). Chromosomes were counterstained with DAPI. Fluorescent-specific signals were visualized under confocal microscope. For every cell preparation, 30 metaphases were scored telomere-free chromosome ends. For karyotyping, metaphase cells were prepared by standard cytogenetics methods. Karyotypes were described according to the International System for Human Cytogenetic Nomenclature [52].

### Immunostaining

Cytospin slides of cells were fixed in 4% paraformaldehyde, permeabilized with PBS containing 0.25% Trition X-100, and blocked with 1% BSA in PBS. Then cells were incubated with primary antibody against Ser139 phospho-H2AX (Cell Signaling #9718) in room temperature for 1 h, and then with goat anti-rabbit IgG (H+L) secondary antibody, Alexa Fluor 488 (Molecular Probes). For each experiment, random view fields were selected and 100 nuclei were scored for each group.

### Realtime qPCR

Human CD34+ HSPC were transduced with AE or control empty vector, GFP+ cells were sorted 4 days after transduction. RNA was isolated using the RNeasy kit (Qiagen), and then reversed transcribed using MuLV Reverse Transcriptase and random hexamers (Applied Biosystems). The cDNA was then subject to qPCR using SYBR Green technology (Roche). Expression level was calculated by ΔΔCt method, normalized to PGK1. Primers are: PGK1 F GGGAAAAGATGCTTCTGGGAA; PGK1 R TTGGAAAGTGAAGCTCGGAAA; hTERT F CCGCTTCCTCAGGAACACCA; hTERT R GCAGCTCGACGACGTACACA.

## SUPPLEMENTARY MATERIAL FIGURE


