# Supplementary material for: Immortalization of human AE pre-leukemia cells by hTERT allows leukemic transformation
Source: Oncotarget. 2016 Aug 5;7(35):55939–50. doi: 10.18632/oncotarget.11093 (PMC5302887; doi:10.18632/oncotarget.11093)
Supplement: Supplementary file 1 [file oncotarget-07-55939-s001.pdf]

Immortalization of human AE pre-leukemia cells by hTERT allows leukemic transformation

Supplementary Material

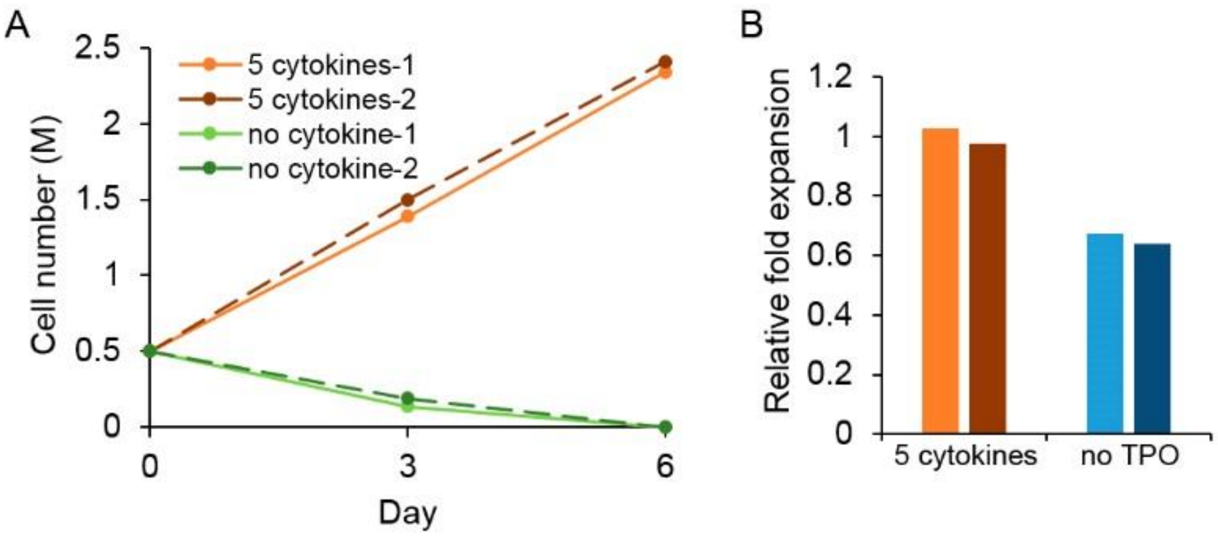

Figure S1. Leukemic transformation of AE-hTERT cells does not lead to cytokine independence. (A) Growth curve of transformed AE-hTERT clone in culture with all 5 cytokines or no cytokines. (B) Relative fold expansion of transformed AE-hTERT cells cultured with all 5 cytokines or without TPO for 4 weeks. Duplicates of the experiment are shown. The mean fold expansion of 5-cytokines sample #1 is set as 1.
